# Comprehensive analysis of age‐related somatic mutation profiles in Chinese young lung adenocarcinoma patients

**DOI:** 10.1002/cam4.1839

**Published:** 2019-03-01

**Authors:** Bo Yang, Jie Li, Fang Li, Hongxia Zhou, Weiwei Shi, Huaiyin Shi, Shengjie Sun, Wending Sun, Jinliang Wang, Junxun Ma, Xiang Yan, Yi Hu, Shunchang Jiao

**Affiliations:** ^1^ Department of Oncology General Hospital of Chinese PLA Beijing China; ^2^ Department of Pathology General Hospital of Chinese PLA Beijing China; ^3^ GenomiCare Biotechnology Co. Ltd. Shanghai China

**Keywords:** age‐related dimorphism, lung adenocarcinoma, oncogenic genetic alterations, whole‐exome sequencing, younger adults

## Abstract

**Background:**

Lung adenocarcinoma in young adults is a rare entity with the oncogenic genetic alterations associated being poorly understood. In the present study, the effect of genetic alterations in lung adenocarcinoma patients diagnosed in young patients is reported.

**Methods:**

Twenty young lung adenocarcinoma patients (age years: median: 33.5, range: 24‐36) were enrolled in the current study and 24 patients who were at common age of the disease onset (age years: median: 61.5, range: 52‐79) were selected for comparison. Paraffin sections of lung adenocarcinoma were analyzed using the whole‐exome sequencing platform.

**Results:**

Similar number of somatic mutations per tumor were found in the young patients and their older counterparts. Although no age‐related differences were detected in the numbers of lung adenocarcinoma patients harboring well‐known gene variants, mutations in *FRG1* and *KMT2C* were associated with a younger age especially after correcting for tobacco smoking and sex (*FRG1*: *P* = 0.027, *KMT2C*: *P* = 0.046). Five genetic variants showed higher alteration frequencies in young patients compared to the unclassified East Asian population, suggesting these mutations as disease‐related hereditary germline variants.

**Conclusions:**

These results suggest different characteristics of lung adenocarcinoma between the young and the patients at common age of onset. Young patients with lung adenocarcinoma have a distinctly unique prevalence of oncogenic genetic alterations.

## 1 INTRODUCTION

Non–small‐cell lung cancer (NSCLC) is widely understood as its heterogeneity, from the profile of both its clinical characteristics and geneticmakeup.^1^ Molecularly targeted therapy has largely revolutionized the treatment of NSCLC in genomically defined subsets of patients.^2^ The identification of specific types of EGFR mutation and ALK fusions classically confer sensitivity to matched therapies and implies a significant survival benefit from approved targeted agents.^3^, ^4^ In comprehensive genomic profiling analysis, driver genetic alterations have been identified in approximately 50% of lung adenocarcinomas, including variants in *EGFR*, *KRAS*, *BRAF*, *HER2*, *RET,* and *ROS1*.^2^, ^5^, ^6^, ^7^

Cancers such as breast cancer, colon cancer, and acute lymphoblastic leukemia are understood to present a distinct disease biology in patients diagnosed at a young age.^1^, ^2^ In contrast, NSCLC in the young is a poorly studied clinical entity.^1^, ^2^, ^8^ It has been reported that 70 years of age was the median age for diagnosis of NSCLC and patients diagnosed younger than 50 years accounted for less than 5% of patients.^1^ In China, the incidence of lung cancer in male and female patients diagnosed before 45 years of age is 1.71% and 1.16%, and diagnosed before 30 years of age is 0.13% and 0.08%.^9^ Recent data have suggested that ALK and ROS1 rearrangement produced a higher incidence in the young NSCLC patients compared with the patients diagnosed at an older age.^1^, ^10^, ^11^, ^12^, ^13^ These lung cancers only represent a small proportion of all NSCLC, and there are many more types of targetable genetic alterations in lung cancer apart from ALK and ROS1 rearrangement.^14^

Currently, studying the genomic variants especially in young patients and their relationship with age remains challenging, due to multiple confounding factors, for example, smoking history and sex, relatively rarity of young lung adenocarcinoma patients (YLAPs), as well as the low incidence of many of these targetable genetic alterations.^1^ Moreover, no previous study has discovered whether or not somatic single‐nucleotide variants (SNVs) significantly differ between YLAPs younger than 36 years of age and those diagnosed at common age of the disease onset (around 70 years of age).

In the present study, we performed analyses for somatic SNVs and driver genomic alterations in both YLAPs and patients diagnosed at common age of lung adenocarcinoma onset. Genetic variants fundamentally associated with a younger age at diagnosis were further investigated with other confounding factors in order to establish more clinically meaningful interpretations.

## 2 MATERIALS AND METHODS

### 2.1 Study design, patient selection, and sample information

The patients eligible for the study were collected from the NSCLC pathology database of our institution. The age at the time of initial diagnosis, smoking history, sex, and the disease stage were obtained from the hospital medical records. In total, 44 lung adenocarcinoma patients were included in the current study. Twenty of these participants were classified as “young” and diagnosed at an age ≤36 years. The other 24 patients were classified as “older” (age at diagnosis >50 years). Samples from lung adenocarcinomas were obtained in the formalin‐fixed paraffin‐embedded form.

### 2.2 Exome capture, library construction, and sequencing

Genomic DNA was fragmented and hybridized to Agilent SureSelect Human All Exome kit V5. Exome‐enriched shotgun libraries were sequenced on the Illumina Xten platform, and pair‐end reads with size of 150 *2 bp were generated. Image analysis and base calling were performed with Illumina CAVSAVR version 1.8, using default parameters. After removing reads with sequence matching, the sequencing adaptors and low‐quality reads with exact match, high‐quality reads were aligned to the NCBI human reference genome hg19 using Burrows‐Wheeler Aligner tools (Figure S2).

### 2.3 Somatic and germline mutation identification

Reads in fastq format were initially processed with Genome Analysis Toolkit (GATK) version 3.5. Localized (insertion‐deletion) indel realignments were performed using GATK. Regions that needed to be realigned were identified using the GATK Realigner Target Creator (Figure S2).

#### 2.3.1 SNV detection

For SNV calling, the MuTect algorithm was applied to identify candidate somatic single‐nucleotide variants in tumor compared with a matched control blood sample from one patient. GATKs HaplotypeCaller was used to call germline SNV mutations via local re‐assembly of haplotypes. SNV annotation was performed using ANNOVAR (Figure S2). To predict the effect nonsynonymous mutations might have on the encoded proteins we used dbNSFP31, which collates the outputs from the prediction programs SIFT32 and Polyphen2.

#### 2.3.2 Indel detection

Tumor samples and matched control blood samples were analyzed with VarScan v2.3.8. Candidate somatic indel were only considered if they were supported by at least five reads and if the number of supporting reads divided by the maximum of the read depth at the left and right breakpoint positions was larger than 0.05. All somatic indel calls were manually reviewed using the Integrative Genomics Viewer. GATKs HaplotypeCaller was used to call germline indel mutations via local re‐assembly of haplotypes. Indels were annotated as described for SNVs (Figure S2).

### 2.4 ALK rearrangement detection with immunohistochemistry (IHC)

ALK IHC was performed on freshly cut 4‐μm thick formalin‐fixed paraffin‐embedded tissue sections using the Ventana ALK (D5F3) CDx assay. The anti‐ALK (D5F3 clone) rabbit monoclonal antibody was applied on a BenchMark XT autostainer with the Ultraview diaminobenzidine detection kit (Ventana Medical Systems Inc, Tucson, AZ, USA).^15^ Staining was interpreted clinically as positive if tumor cells showed a moderate or strong multifocal or diffuse expression. All positive cases showed a granular cytoplasmic pattern.

### 2.5 Analysis of mutation frequency and mutation spectrum

The mutation frequency was analyzed by counting the number of ALK rearrangements detected with IHC as well as the number of variants annotated by ANNOVAR from WES data. To analyze the mutation spectrum, SNVs processed with MuTect in all sequenced regions (not limited to coding regions) were analyzed.

### 2.6 Statistical analysis

The chi‐squared and Fisher's exact tests were used to investigate differences in categorical variables (eg, sex, smoking history, and genetic variants) between the young and older patient subgroups in unadjusted analyses. Binomial logistic regression analyses were carried out to correct for important covariates such as sex and the smoking history, in adjusted analyses. For investigating whether or not age significantly associated with mutations occurred in lung adenocarcinoma patients, the impact with a *P*‐value <0.1, detected in unadjusted analyses, was further tested with adjusted analyses. A *P*‐value <0.05 was considered significant in both unadjusted and adjusted analyses. The statistical analyses were performed using SPSS software (Version 23.0.0, IBM corp., Armonk, NY).

## 3 RESULTS

### 3.1 Patient characteristics

Twenty East Asian young adult patients who had lung cancer diagnosed as adenocarcinoma before 36 years of age were enrolled in the current study. Twenty‐four patients diagnosed at the common age of the disease onset were selected for comparison and identified as the older counterparts. The demographics of all 44 patients with adenocarcinoma are listed in Table 1. Of the young cohort of patients, 10 (50.0%) were males, 15 patients (75.0%) never smoked, and their median age was 33.5 years (range, 24‐36). Among the older patients, 12 (50.0%) were males, 16 patients (66.7%) never smoked, and the median age was 61.5 years (range, 52‐79). Smoking history (*P = *1.00) or sex (*P = *1.00) did not significantly differ between the young and older groups of patients. In young patients, there were 14 (70.0%), 2 patients (10.0%) and 4 patients (20.0%) with stage I disease, stage II disease, and stage III disease, respectively. No young patients were at stage IV. The disease stage information of older patients was available in 5 out of 24 participants, where three patients (12.5%) were in stage III and two patients (8.3%) in disease stage IV (Table 1).

**Table 1:** Characteristics of young adult (age ≤ 36 y, median: 33.5) and older (age > 50 y, median: 61.5) lung adenocarcinoma individuals

Characteristics	Age ≤ 36 y n = 20, Median = 33.5	Age > 50 y n = 24, Median = 61.5	*P*‐value
	No. of cases (%)	No. of cases (%)	
Sex			
Male	10 (50.0%)	12 (50.0%)	1
Female	10 (50.0%)	12 (50.0%)	
Histological classification			
Adenocarcinoma in situ	2(10%)	NA	NA
Invasive adenocarcinoma	6 (30%)	NA	
Mucinous adenocarcinoma	2 (10%)	NA	
Unclassified adenocarcinoma	10(50%)	NA	
Stage			
I	14(70.0%)	NA	NA
II	2(10.0%)	NA	
III	4(20.0%)	3	
VI	0 (0%)	2	
Smoking history			
Always smoker	5 (25.0%)	5 (20.8%)	1
Never smoker	15 (75.0%)	16 (66.7%)	
Unknown	0 (0%)	3 (8.3%)	
*EGFR* mutation	7 (35.0%)	14 (58.3%)	0.125
*ALK *arrangement	2 (25.0%, n = 8)	0 (0%, n = 7)	0.509
*KRAS *mutation	0 (0%)	2 (8.3%)	0.552
*TP53 *mutation	7 (35.0%)	10 (41.7%)	0.888

*P*‐value: *P*‐values representing the differences of sex, histologic classification, disease stage, tobacco smoking history, whether or not carrying the mutations in *EGFR*, *KRAS* and/or, *TP53* or the *ALK* arrangement between young (age ≤ 36 y) and older (age > 50) patients were obtained using chi‐squared tests (2‐sided) or Fisher's exact test where appropriate. NA: data not applicable.

### 3.2 Mutation frequency and mutation spectrum

The mutation frequency and mutation spectrum of 44 samples were analyzed from processed WES data (Figure S1). As illustrated in the Figure S1, the median number of somatic mutations per tumor was 92 in the young patients and 84 in the older patients. No significant difference was detected with regard to the numbers of somatic mutations between two groups (*P = *0.428, Figure S1).

According to the mutation spectrum, frequencies (*P = *0.730, median: young/old = 11/13.5) or percentage (*P = *0.935, mean: young/old = 39.52%/39.24%) of C:G‐>A:T nucleotide substitutions did not significantly differ between young and older lung adenocarcinoma patients (Figure S1).

### 3.3 Associations between age and prevalence of *EGFR*, *ALK*, *KRAS,* and *TP53* in lung adenocarcinoma

As shown in Table 1, among the eight young and seven older adenocarcinoma patients with available data about *ALK* translocations, two patients (25%) and no one were identified as *ALK* translocation carriers, respectively. *EGFR*, *KRAS*, and *TP53* mutations were detected in 7 (35.0%), 0 (0%), and 7 (35.0%) patients in 20 young patients, and were respectively detected in 14 (58.3%), 2 (8.3%), and 10 (41.7%) older patients out of 24 (Table 1). Although patients with lung adenocarcinoma diagnosed at a younger age showed a lower incidence and percentage of *EGFR* mutations than patients diagnosed at the common age of onset, young and older patients did not demonstrate significant differences in the prevalence of *ALK* translocations and mutations in *EGFR*, *KRAS*, or *TP53* (*ALK*: *P = *0.509, *EGFR*: *P = *0.125, *KRAS*: *P = *0.552, *TP53*: *P = *0.888, Table 1).

### 3.4 Associations between age and prevalence of other gene variants in lung adenocarcinoma

**Figure 1:**
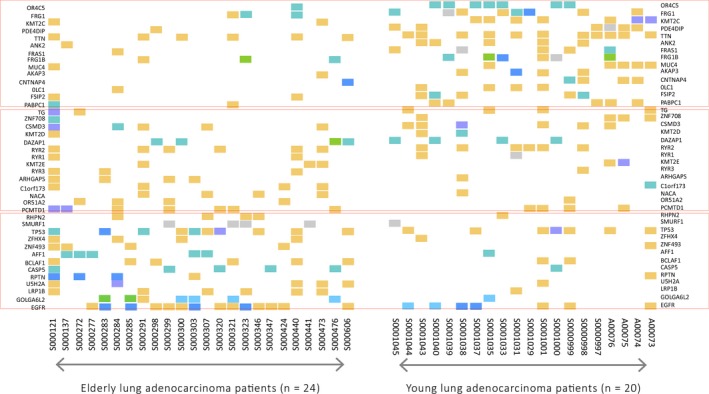
Heat‐map representing genetic events in young and/or older lung adenocarcinoma patients. Heat‐map of genetic events in 20 young and 24 older patients’ lung adenocarcinoma samples. Events including missense variants, stop‐gained variants, frameshift variants, (disruptive) inframe deletion, (disruptive) inframe insertion, and splice region/donor/acceptor variant. The distinct types of variations are colored according to the legend provided

As illustrated in Figure 1, mutations in genes OR4C5, FRG1, KMT2C, PDE4DIP, TTN, ANK2, FRAS1, FRG1B, MUC4, AKAP3, CNTNAP4, DLC1, FSIP2, and PABPC1 more commonly occurred in YLAPs (Tables S1 and S2). Older lung adenocarcinoma patients carried more mutations in the genes SMORF1, TP53, ZFHX4, ZNF493, AFF1, BCLAF1, CASP5, RPTN, USH2A, LRP1B, GOLGA6L2, and EGFR than YLAPs (Figure 1, Tables S1 and S2). Mutations in genes TG, ZNF708, CSMD3, KMT2D, DAZAP1, RYR2, RYR1, KMT2E, RYR3, ARHGAP5, C1orf173, NACA, OR51A2, PCMTD1, and RHPN2 did not present predisposition to age (Figure 1, Tables S1 and S2).

The age‐related association was identified as top‐rank with mutations occurring in two genes FRG1 and KMT2C *FRG1* mutations were detected in 8 (40.0%) young and 3 (12.5%) older lung adenocarcinoma patients (*P = *0.081) (Table 2). *FRG1* is a subtelomeric gene encoding the protein associated with telomere length. Similarly, genetic alterations in *KMT2C* were found in 7 (35.0%) of young and 2 (8.3%) of older lung adenocarcinoma patients (*P = *0.05727) (Table 2). *KMT2C* (or *MLL3*) is encoding the protein that belongs to the chromatin‐modifying proteins and implements the histone H3 lysine 4 monomethylation on enhancers, and is important for the transition from inactive “poised” enhancers to active enhancers. Variants in these two genes were associated with a younger age (*FRG1*: *P = *0.029, *KMT2C*: *P = *0.081) and both suggested enhanced association after correcting for tobacco smoking and sex (*FRG1*: *P = *0.027, *KMT2C*: *P = *0.046) (Table 3). Interestingly, the impact of *KMT2C* was strengthened after taking *KMT2D *and *KMT2E* into consideration. YLAPs have a significantly higher possibility of carrying mutation(s) in at least one of the three KMT2 family genes—*KMT2C*, *KMT2D, *and *KMT2E* (*P = *0.042) (Table 2).

**Table 2:** Unadjusted analyses testing differences in gene mutation frequencies between young adults and older lung adenocarcinoma patients

Gene	Young, n (%) n *=* 20	Older, n (%) n *= *24	*P*‐value (Chi‐squared test or Fisher's exact test)
*FRG1*	8 (40.0)	3 (12.5)	0.0805
*KMT2C* (*MLL3*)	7 (35.0)	2 (8.3)	0.0573
*KMT2D* (*MLL4*)	2 (10.0)	1 (4.2)	0.583
*KMT2E* (*MLL5*)	2 (10.0)	3 (12.5)	1
*KMT2C*/*KMT2D*/*KMT2E*	10 (50.0)	4 (16.7)	**0.042** a

*KMT2C*/*KMT2D*/*KMT2E*: represent numbers of individuals carrying mutations in at least one of the genes of KMT2C, KMT2D, and KMT2E.

*P*‐value: *P*‐values representing the differences of mutation frequencies in *FRG1*, *KMT2C*/*KMT2D*/*KMT2E* between young (aged ≤ 36 y) and older (aged > 50 y) patients were obtained using chi‐squared tests (2‐sided); *P*‐values representing the differences of mutation frequencies in *KMT2C* (*MLL3*), *KMT2D* (*MLL4*), *KMT2E* (*MLL5*) between young (age ≤ 36 y) and older (age > 50 y) patients were obtained using Fisher's exact tests. *P* **< 0.05 was considered to be statistically significant and shown in bold.

a *P* < 0.05.

**Table 3:** Logistic regression analyses investigating the association of gene alterations with age after the correction of potential predictors including sex and tobacco smoking

The presence of mutations	Characteristics	Odds ratio	95% CI	*P*‐value
*FRG1*	*Model 0*			
	Age	0.944	0.896‐0.994	**0.029** a
	*Model 1*			
	Age	0.932	0.875‐0.992	**0.026** a
	Tobacco smoking	0.267	0.027‐2.679	0.262
	*Model 2*			
	Age	0.932	0.875‐0.992	**0.027** a
	Tobacco smoking	0.271	0.020‐3.691	0.327
	Sex	0.980	0.157‐6.102	0.983
*KMT2C*	*Model 0*			
	Age	0.954	0.904‐1.006	0.081
	*Model 1*			
	Age	0.933	0.872‐0.998	**0.044** a
	Tobacco smoking	2.982	0.475‐18.716	0.244
	*Model 2*			
	Age	0.933	0.872‐0.999	**0.046** a
	Tobacco smoking	2.383	0.247‐22.993	0.453
	Sex	1.410	0.173‐11.500	0.749
*KMT2C/KMT2D/KMT2E*	*Model 0*			
	Age	0.961	0.920‐1.003	0.069
	*Model 1*			
	Age	0.949	0.903‐0.998	**0.0403** a
	Tobacco smoking	3.904	0.750‐20.317	0.1055
	*Model 2*			
	Age	0.950	0.904‐0.998	**0.041** a
	Tobacco smoking	3.217	0.425‐24.346	0.258
	Sex	1.333	0.224‐7.932	0.752

*KMT2C*/*KMT2D*/*KMT2E*: represent individuals carrying mutations in at least one of the genes of *KMT2C*, *KMT2D,* and *KMT2E*.

Model 0: models adjusted for age. Model 1: models adjusted for age and smoking history. Model 2: models adjusted for age, smoking history, and sex.

*P*‐values were obtained using binomial logistic regression analyses. *P*‐values < 0.05 were considered to be statistically significant and set in bold.

a *P* < 0.05.

No significant association was detected between gene alterations mentioned above and smoking history per se, except that never smokers demonstrated relatively lower mutation frequencies in gene KMT2E (*P = *0.069) (data were not shown in the tables). Moreover, genetic alterations in *EGFR *(*P = *0.417)*, KRAS* (*P = 0.118*) *or ALK* (*P = *0.826) were not associated with tobacco smoking (Table 1).

### 3.5 Pathogenic germline mutations in genes TP53, TGFBR2, MLH3, and ELAC2 detected in YLAPs

Among 20 YLAPs, one (ID: S0001040) showed a pathogenic germline variant (p.R141H) in *TP53 *(alteration frequency: 5% in YLAPs vs <0.01% in East Asian (EAS)). A germline variant (p.V741F) in *MLH3* was detected in 3 out of 20 young patients (ID: S0000998, S0001037, A00075) (alteration frequency: 10% in YLAPs vs 1.18% in EAS). Two out of 20 individuals (ID: S0001031, S0001043) showed a pathogenic germline mutation (p.T315M) in *TGFBR *(alteration frequency: 10% in YLAPs vs 1.48% in EAS). Two pathogenic germline alterations (p.A501T and p.S217L) in *ELAC2* were detected in a YLAP (ID: S0001029) (p.A501T: alteration frequency: 5% in YLAPs vs 0.70% in EAS; p.S217L: alteration frequency: 5% in YLAPs vs 3.55% in EAS). All the above genetic mutations occurred highly in the YLAPs in comparison with the unclassified EAS population (according to ANNOVAR filter‐based annotation table esp6500siv2_all (Build: hg19, Date: 20141222)).

## 4 DISCUSSION

In the current study, we established that genetic variants associated with lung adenocarcinoma differed between patients diagnosed when young or diagnosed at the common age of onset. To our knowledge, this is the first study that identifies *FRG1* and *KMT2C* (*MLL3*) as susceptibility genes for the pathogenesis of lung adenocarcinoma exclusively among patients diagnosed younger than 36 years of age.

We detected that YLAPs and patients diagnosed older than 50 years harbored similar numbers of somatic mutations per tumor, although the tumor mutational burden has been suggested to be increased with age across cancer types in some studies.^16^ Meanwhile, five pathogenic germline variants in four genes (including well‐studied lung cancer associated genes TP53 and MLH3) showed fundamentally higher occurrence frequencies in YLAPs in comparison with the unclassified EAS population, but none of these variants has been detected in the older group patients. Tanaka et al^2^ proved that in YLAPs (age <40 years), 30% had *EGFR* mutations. The prevalence was consistent with our findings where distinct types of genetic alterations in *EGFR* were detected in 7 out of 20 patients (35%) diagnosed with lung adenocarcinoma who were younger than 36 years. *ALK* translocations were shown in 2 out of 8 young patients in our study. The prevalence was relatively lower than that in other studies, for instance the *ALK* translocations were positive in 42%^5^, ^12^ of young lung cancer patients as shown by Nagashima et al and were positive in 41% (33 of 81) of adenocarcinoma patients under 40 years as illustrated by Tanaka et al.^2^, ^12^ It has been proved that ALK translocations are significantly higher in young patients with stage IV adenocarcinoma than in those with stage I through III adenocarcinoma,^2^ and in our study all 20 young patients were in stage I through III, and 14 were in stage I. We demonstrated that the variants in *KRAS* were more frequent in patients (8.3%) diagnosed at common age of onset than that in patients diagnosed before 36 years of age (0%). The same trend has been shown in other studies, for instance *KRAS* mutations were less frequent in the younger population as shown by Tanaka et al (2% vs 10%), as well as by Sacher et al.^1^, ^2^, ^17^ In our analysis, no fundamental difference was shown with regard to the frequency of alterations in *TP53* between young and older patients, which is in agreement with previous studies, which did not detect genetic variants of *TP53* in 16 out of 17 young (age range: 25‐41 years) and 10 out of 11 older (age range: 68‐82 years) NSCLC patients.^17^

Genetic alterations in *FRG1* and *KMT2C* (*MLL3*) were both significantly associated with a younger age in patients diagnosed with lung adenocarcinoma, especially after correcting for potential predictors, for example, sex and smoking history.

**Figure 2:**
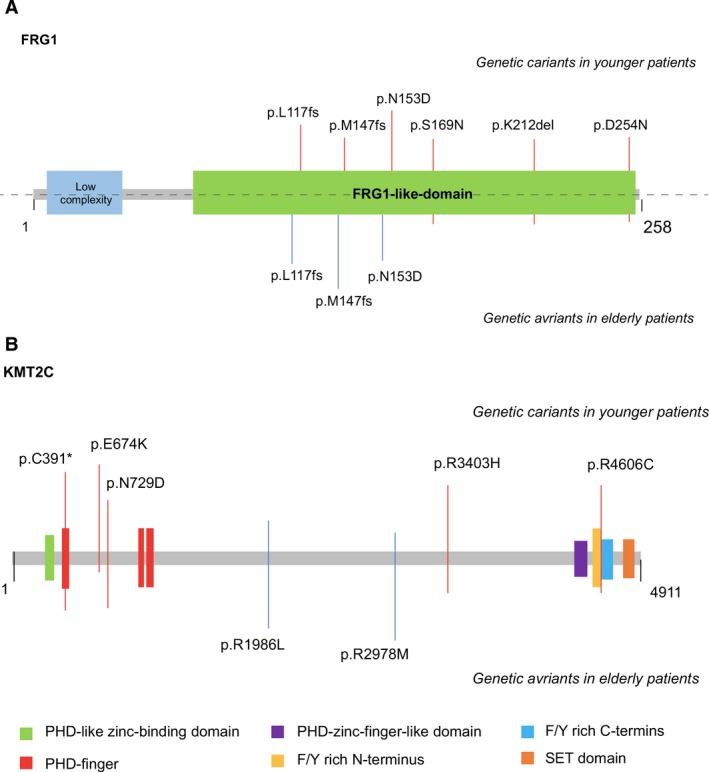
Localizations of mutations in FRG1 and KMT2C. The amino acid position of each FRG1 and KMT2C mutation is depicted relative to the open reading frame of the gene, along with the position of known protein domains. A, Genetic variants detected in *FRG1*. *FRG1* mutations shown in younger patients include p.L117fs, p.M147fs, p.N153D, p.S169N, p.K212del, and p.D254N. In older patients, genetic variants in *FRG1* include p.L117fs, p.M147fs, and p.N153D; B, Genetic variants detected in *KMT2C*. In younger patients, *KMT2C* mutations include p.C391*, p.E674K, p.N729D, p.R3403H, and p.R4606C. In older patients, genetic variants in *KMT2C* include p.R1986L and p.R2978M

*FRG1* is a gene that has been proven to be associated with the disease of facioscapulohumeral muscular dystrophy (FSHD).^18^ FRG1 is located in the region close to a large array of repetitive sequences (D4Z4), which possess characteristics of CpG islands.^19^ The complex epigenetic mechanisms that occur in this region results in the perturbation of heterochromatic gene silencing in the subtelomeric domain of the long arm of chromosome 4 and further plays a significant role in the onset and development of FSHD.^20^ The relationship between genetic alterations in *FRG1* and the prevalence of lung adenocarcinoma has not been shown in previous studies even after taking age into consideration. However, global DNA hypomethylation has been recognized as a key epigenetic change in lung adenocarcinoma, inducing chromosomal instability and aberrant gene expression through alterations in the methylation levels in promoter CpG islands.^21^ As shown in Figure 2A, FRG1 variants detected in young and older lung adenocarcinoma patients were both located in the region of the FRG1‐like domain. All three types of variations shown in the patients diagnosed at common age of onset can also be seen in younger patients (Figure 2A).

*KMT2C* (*MLL3*) maps to chromosome 7q36.1 and encodes a protein predicted with a length of 4911 amino acids.^22^ *MLL3* contains two plant homeodomains (PHD), a suppressor of variegation, enhancer of zeste and trithorax (SET) and two phenylalanine tyrosine (FY)—rich domains (Figure 2B).^23^ It has been proven that the PHD and SET protein domains act as chromatin regulators and are altered in distinct types of cancers.^23^ *MLL3*, as part of a transcriptional coactivator complex, is a tumor suppressor involved in a number of cellular processes, including regulation of homeostasis and hormone receptor signaling.^24^, ^25^, ^26^ *MLL3* mutations have been reported in 14% (98/702) of lung adenocarcinoma samples analyzed in the Catalogue of Somatic Mutations in Cancer (COSMIC) database (Oct 2017). According to previous studies, variants in *MLL3* are frequently deleted in myeloid leukemia. The inactivating mutations of *MLL3* have been shown in colorectal cancer and medulloblastoma,^27^, ^28^ and its somatic alterations have also been reported in pancreatic ductal adenocarcinoma and glioblastoma.^29^ The expression level of *MLL3* decreased in primary breast tumor samples and esophageal cancer cell lines,^30^, ^31^ suggesting that *MLL3* functions as a tumor suppressor gene in cancer development.^22^ As shown in Figure 2B, only young but not older lung adenocarcinoma patients carried *MLL3* mutations in the functional motif regions of this gene, for instance the PHD‐finger regions and F/Y rich C‐terminus. The *MLL3* gene variant p.C391*, which is located at one of the PHD‐finger regions (Figure 2B), was detected in 3 out of 20 YLAPs (15%) in our study. This alteration is expected to truncate the MLL3 protein at amino acid 391 out of 4911, resulting in the loss of the majority of the protein, including the FYR domains and the SET domain (UniProt, Figure 2B). Truncation of the SET domain has been shown to disrupt gene regulation and result in widespread histone methylation disturbances.^32^ Histone methylation is one type of epigenetic modifications known to reflect the vital cellular changes in the individuals with lung cancer.^33^ It is well known that the epigenome serves as an interface between the environment and the genome.^34^

In conclusion, we analyzed age‐related genetic alterations in the patients diagnosed with lung adenocarcinoma before 36 years of age and in the patients diagnosed at the common age of onset (age years in our study: median: 61.5, range: 52‐79). Significant differences were detected with regard to the occurrence of both somatic and germline mutations between the young and the older patients, regardless of tobacco smoking history and sex. As far as we know, no previous study has described the gene mutation characteristics especially gene mutation burden of lung adenocarcinoma among patients younger than 36 years of age, and our study has filled this gap. Further perspective studies should elucidate why specific mutations discussed in our study observed for the YLAPs, as well as identify therapeutic strategies in this subgroup of patients.

## ETHICAL APPROVAL

All procedures performed in studies involving human participants were in accordance with the ethical standards of the institutional and/or national research committee and with the 1964 Helsinki declaration and its later amendments or comparable ethical standards. Informed consent was obtained from all individual participants included in the study.

## CONFLICT OF INTEREST

The authors declare that they have no competing interests.
