# Supplementary figures and images for: Comprehensive analysis of age‐related somatic mutation profiles in Chinese young lung adenocarcinoma patients
Source: Cancer Med. 2019 Mar 1;8(4):1350–8. doi: 10.1002/cam4.1839 (PMC6488136; doi:10.1002/cam4.1839)

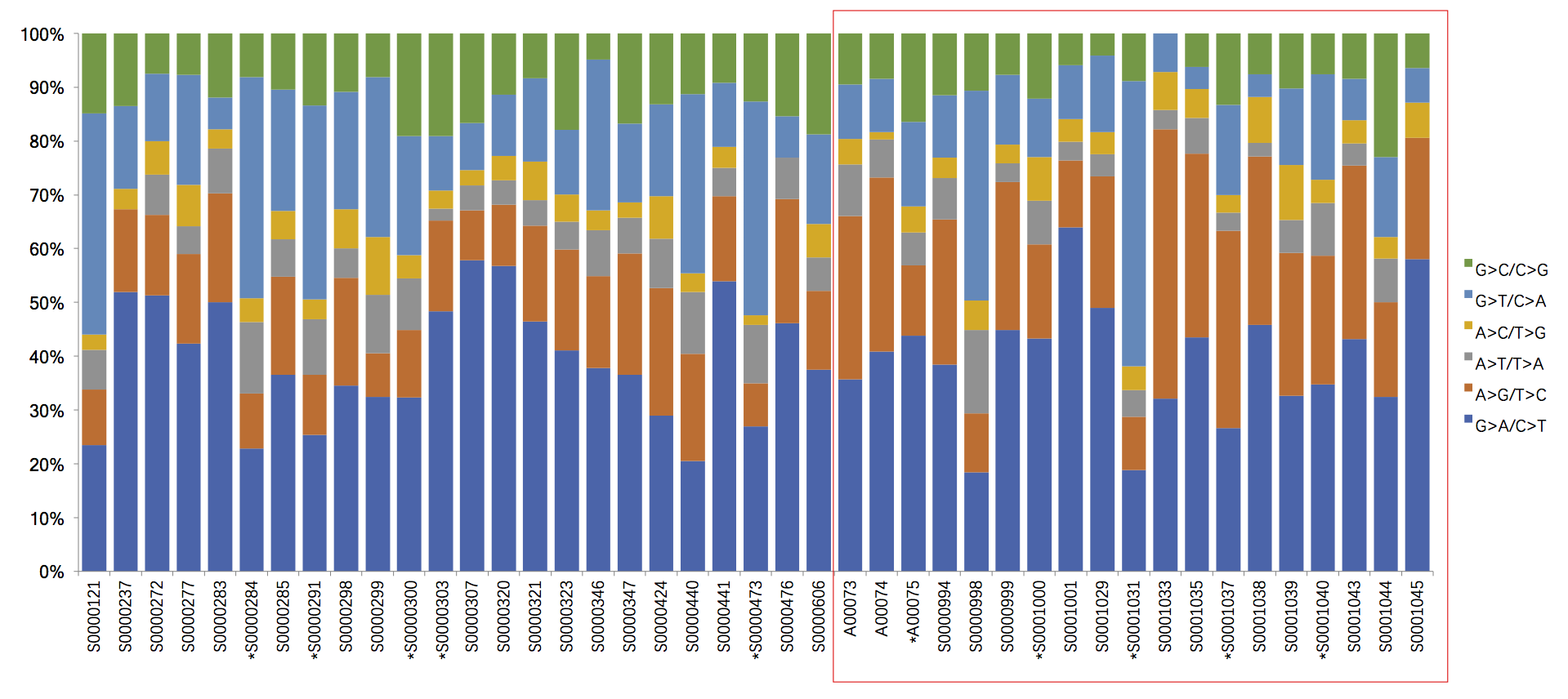

Supplement: Supplementary file 1 [file CAM4-8-1350-s001.tiff]

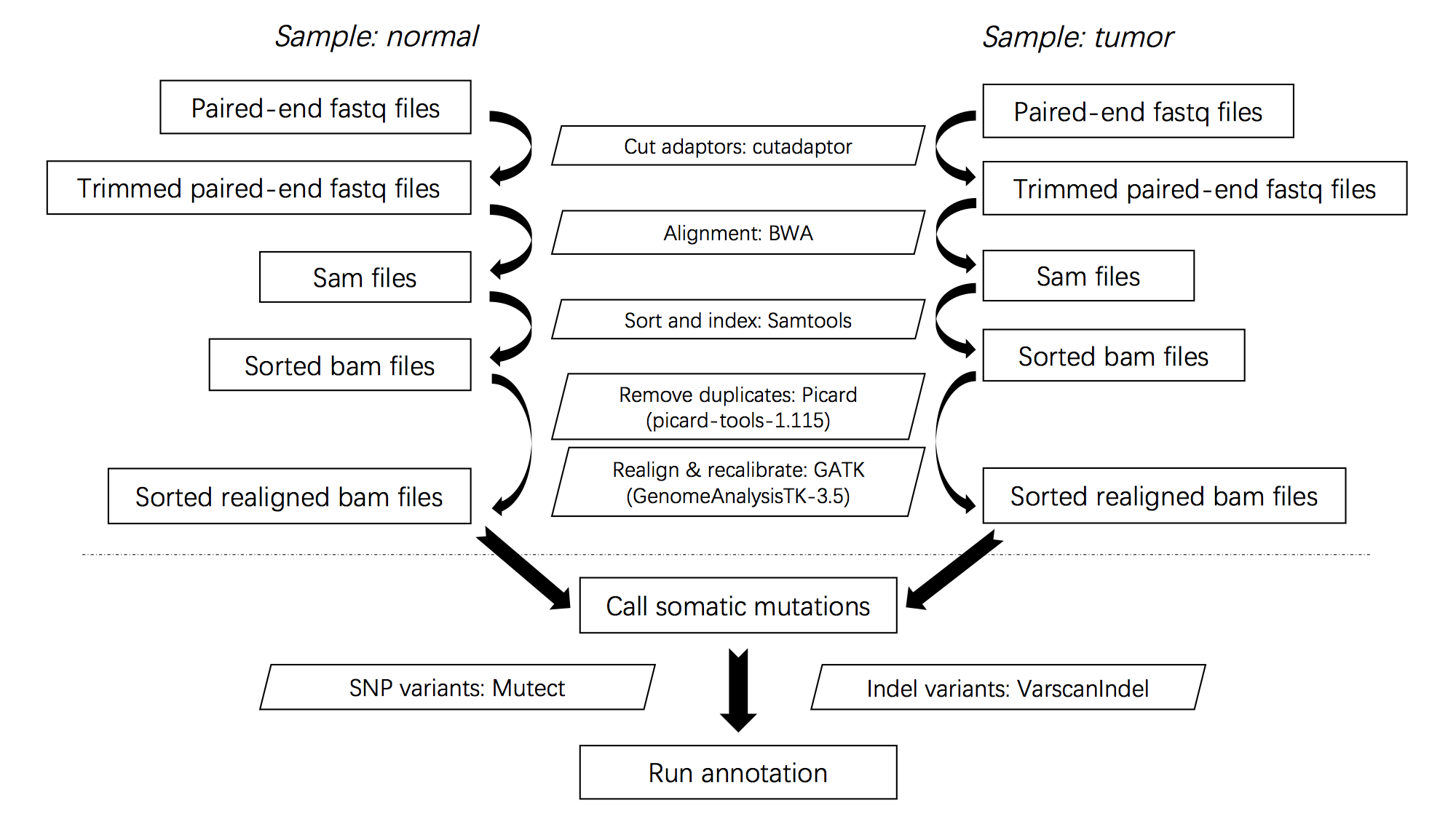

Supplement: Supplementary file 2 [file CAM4-8-1350-s002.tiff]
