# Supplementary material for: Comprehensive analysis of age‐related somatic mutation profiles in Chinese young lung adenocarcinoma patients
Source: Cancer Med. 2019 Mar 1;8(4):1350–8. doi: 10.1002/cam4.1839 (PMC6488136; doi:10.1002/cam4.1839)
Supplement: Supplementary file 3 [file CAM4-8-1350-s003.docx]

Supplementary Figure 1. Mutation Spectrum Representing Base Substitutions in Younger and Elderly Lung Adenocarcinoma Patients

A stacked bar graph representing the frequency of each type of base substitution for all somatic point mutations in exomes of 20 young patients (age < 40 years) and 24 elderly patients (age > 50 years). Bars representing base substitution of younger individuals were rounded with a red square. Individuals with a smoking history were labeled together with the sign “*”.

Supplementary Figure 2. Flow chart illustrate the process for performing next generation sequencing tests staring from fastq files to annotated variant call format (VCF) document

In the current study, sequencing data collected from normal and tumor samples were both analyzed from the paired-end fastq files. The 3’ adapters and 5’ adaptors were firstly trimmed by using the software package named “Cutadapt”. The trimmed paired-end fastq data were than processed by using the Burrows-Wheeler-Alignment (BWA) mem algorithm tool (version: bwa-0.7.12), and Sequence Alignment/Map (SAM) format files were achieved in this step for both normal and tumor samples. The sort and index algorithm in the SAMtools software package were than conducted for analyzing, editing and transforming SAM files into BAM files, which are the compressed binary representation of the SAM files. The NGS software Tools Picard (picard-tools-1.115) and GenomeAnalysisTK-3.5 (GATK) were applied for further processing the BAM files, including removing duplicates, realignment and recalibration. Afterwards, the sorted realigned bam files from both normal and tumor samples were analyzed together to perform the somatic mutation calling, where the single nucleotide polymorphisms (SNPs) and Indel variants were respectively identified through applying the MuTect (mutect-1.1.7) and the VarScan (VarScan.v2.3.9) algorithm. The VCF files were obtained in the last step showing the somatic alterations and the annotation process was further conducted for both SNP and Indel variants.

Supplementary Table 1. Genetic variants detected in young lung adenocarcinoma patients.

Patient ID (patient_ID), chromosome number (chr), position number (position), reference allele (ref_allele), altered allele (alt_allele), the percentage of variant (fre), total sequencing depth in the corresponding genetic position (dp), detected sequencing depth of the variant allele (alt_dp), the gene where the variant locate (gene) and the corresponding amino acid alteration (mutation_p) are provided.

| **patient_ID** | **chr** | **position** | **ref_allele** | **alt_allele** | **fre** | **dp** | **alt_dp** | **gene** | **mutation_p** |
| --- | --- | --- | --- | --- | --- | --- | --- | --- | --- |
| A00073 | chr1 | 152127999 | G | T | 6.45% | 62 | 4 | RPTN | p.P526T |
| A00073 | chr1 | 152128013 | A | G | 7.25% | 69 | 5 | RPTN | p.F521S |
| A00073 | chr1 | 152129115 | T | C | 6.82% | 88 | 6 | RPTN | p.R154G |
| A00073 | chr2 | 179436797 | G | A | 11.54% | 26 | 3 | TTN | p.R24688C |
| A00073 | chr3 | 195505829 | G | A | 20.00% | 15 | 3 | MUC4 | p.P4208S |
| A00073 | chr3 | 195511877 | G | A | 34.38% | 32 | 11 | MUC4 | p.P2192S |
| A00073 | chr3 | 195512287 | G | A | 26.67% | 15 | 4 | MUC4 | p.S2055F |
| A00073 | chr6 | 136594292 | T | C | 23.08% | 26 | 6 | BCLAF1 | p.N629S |
| A00073 | chr7 | 55249005 | G | T | 47.80% | 205 | 98 | EGFR | p.S768I |
| A00073 | chr7 | 55249022 | G | A | 43.25% | 252 | 109 | EGFR | p.V774M |
| A00073 | chr7 | 151962134 | G | T | 6.82% | 88 | 6 | KMT2C | p.C391* |
| A00073 | chr7 | 152007139 | C | T | 9.38% | 32 | 3 | KMT2C | p.R254H |
| A00073 | chr8 | 134125688 | G | A | 11.54% | 26 | 3 | TG | p.R2532Q |
| A00073 | chr17 | 7578466 | G | T | 37.06% | 313 | 116 | TP53 | p.T155N |
| A00073 | chr19 | 21476227 | T | C | 6.12% | 49 | 3 | ZNF708 | p.N514S |
| A00073 | chr19 | 21588020 | A | T | 5.26% | 57 | 3 | ZNF493 | p.K40N |
| A00073 | chr19 | 21607187 | A | G | 13.33% | 30 | 4 | ZNF493 | p.K576E |
| A00073 | chr19 | 21607503 | A | G | 9.09% | 55 | 5 | ZNF493 | p.E681G |
| A00073 | chr20 | 29632643 | T | C | 14.29% | 35 | 5 | FRG1B | p.L153P |
| A00073 | chr1 | 75038438 | CT | C | 5.26% | 57 | 3 | C1orf173 | p.Glu986fs |
| A00074 | chr1 | 144868157 | G | A | 6.25% | 48 | 3 | PDE4DIP | p.P1897L |
| A00074 | chr3 | 195509663 | G | A | 16.67% | 18 | 3 | MUC4 | p.P2930S |
| A00074 | chr3 | 195511273 | G | A | 17.24% | 29 | 5 | MUC4 | p.A2393V |
| A00074 | chr3 | 195511877 | G | A | 19.05% | 21 | 4 | MUC4 | p.P2192S |
| A00074 | chr4 | 190878577 | A | G | 5.23% | 153 | 8 | FRG1 | p.N153D |
| A00074 | chr7 | 151962134 | G | T | 6.19% | 210 | 13 | KMT2C | p.C391* |
| A00074 | chr7 | 151962168 | C | A | 5.23% | 325 | 17 | KMT2C | p.R380L |
| A00074 | chr8 | 101719201 | A | G | 10.08% | 119 | 12 | PABPC1 | p.I454T |
| A00074 | chr8 | 101721899 | C | A | 5.26% | 57 | 3 | PABPC1 | p.E345* |
| A00074 | chr16 | 76572157 | G | A | 5.17% | 58 | 3 | CNTNAP4 | p.R1050Q |
| A00074 | chr20 | 29632643 | T | C | 12.10% | 157 | 19 | FRG1B | p.L153P |
| A00074 | chr8 | 101721932 | CT | C | 8.77% | 57 | 5 | PABPC1 | p.Phe335fs |
| A00074 | chr20 | 29625925 | C | CTTG | 7.64% | 157 | 12 | FRG1B | p.Val58dup |
| A00075 | chr1 | 144952207 | C | T | 9.38% | 32 | 3 | PDE4DIP | p.R308K |
| A00075 | chr2 | 179419267 | C | T | 10.00% | 30 | 3 | TTN | p.E29603K |
| A00075 | chr2 | 179457240 | G | T | 11.11% | 27 | 3 | TTN | p.A19831E |
| A00075 | chr2 | 179515487 | G | A | 9.38% | 32 | 3 | TTN | p.P13234S |
| A00075 | chr3 | 195508108 | G | A | 26.67% | 15 | 4 | MUC4 | p.S3448L |
| A00075 | chr3 | 195509879 | A | G | 12.00% | 25 | 3 | MUC4 | p.S2858P |
| A00075 | chr3 | 195511780 | G | A | 15.38% | 26 | 4 | MUC4 | p.P2224L |
| A00075 | chr3 | 195512456 | T | G | 30.00% | 40 | 12 | MUC4 | p.T1999P |
| A00075 | chr3 | 195513515 | C | T | 10.00% | 30 | 3 | MUC4 | p.A1646T |
| A00075 | chr3 | 195513812 | T | A | 44.44% | 9 | 4 | MUC4 | p.T1547S |
| A00075 | chr3 | 195516676 | G | A | 5.36% | 56 | 3 | MUC4 | p.T592M |
| A00075 | chr7 | 104731836 | G | A | 8.00% | 50 | 4 | KMT2E | p.W599* |
| A00075 | chr8 | 52733079 | A | C | 20.83% | 24 | 5 | PCMTD1 | p.S302R |
| A00075 | chr16 | 76509894 | C | T | 9.09% | 33 | 3 | CNTNAP4 | p.H575Y |
| A00075 | chr17 | 7577120 | C | T | 62.50% | 8 | 5 | TP53 | p.R273H |
| A00075 | chr19 | 21476281 | A | C | 11.43% | 35 | 4 | ZNF708 | p.I496R |
| A00075 | chr19 | 21606831 | A | G | 12.00% | 25 | 3 | ZNF493 | p.E457G |
| A00075 | chr1 | 237777402 | C | CT | 8.82% | 34 | 3 | RYR2 | p.Arg1657fs |
| A00075 | chr3 | 195512373 | G | GGAT | 13.04% | 23 | 3 | MUC4 | p.Ser2026dup |
| A00076 | chr2 | 179404373 | C | T | 12.50% | 24 | 3 | TTN | p.D32807N |
| A00076 | chr3 | 195508133 | G | A | 11.43% | 35 | 4 | MUC4 | p.P3440S |
| A00076 | chr3 | 195509879 | A | G | 8.51% | 47 | 4 | MUC4 | p.S2858P |
| A00076 | chr3 | 195509924 | A | T | 8.57% | 35 | 3 | MUC4 | p.S2843T |
| A00076 | chr3 | 195511142 | T | C | 31.91% | 47 | 15 | MUC4 | p.N2437D |
| A00076 | chr7 | 104742411 | C | T | 8.33% | 36 | 3 | KMT2E | p.R656W |
| A00076 | chr7 | 151945499 | C | T | 10.00% | 30 | 3 | KMT2C | p.E674K |
| A00076 | chr8 | 101719201 | A | G | 9.52% | 42 | 4 | PABPC1 | p.I454T |
| A00076 | chr8 | 114389005 | C | T | 9.38% | 32 | 3 | CSMD3 | p.C7Y |
| A00076 | chr17 | 7578413 | C | A | 22.89% | 367 | 84 | TP53 | p.V173L |
| A00076 | chr19 | 21476951 | C | T | 7.27% | 55 | 4 | ZNF708 | p.V273I |
| A00076 | chr19 | 21476980 | C | T | 12.77% | 47 | 6 | ZNF708 | p.R263Q |
| A00076 | chr19 | 21477005 | C | T | 6.67% | 45 | 3 | ZNF708 | p.E255K |
| A00076 | chr19 | 21477016 | A | G | 12.20% | 41 | 5 | ZNF708 | p.L251P |
| A00076 | chr20 | 29632643 | T | C | 20.00% | 35 | 7 | FRG1B | p.L153P |
| A00076 | chr4 | 79461830 | T | TA | 9.09% | 33 | 3 | FRAS1 | p.Asp3865fs |
| A00076 | chr8 | 101724623 | C | CA | 9.09% | 33 | 3 | PABPC1 | p.Glu313fs |
| A00076 | chr8 | 101724625 | CTT | C | 9.09% | 33 | 3 | PABPC1 | p.Lys312fs |
| A00076 | chr20 | 29625924 | A | ACTT | 5.26% | 57 | 3 | FRG1B | p.Leu57dup |
| A00076 | chr20 | 29632611 | AAAG | A | 11.54% | 26 | 3 | FRG1B | p.Lys144del |
| S0000994 | chr1 | 145015877 | G | T | 19.35% | 93 | 18 | PDE4DIP | p.L142I |
| S0000994 | chr2 | 179544077 | G | A | 6.00% | 50 | 3 | TTN | p.P11244L |
| S0000994 | chr8 | 101719201 | A | G | 7.69% | 52 | 4 | PABPC1 | p.I454T |
| S0000998 | chr1 | 237843774 | G | T | 40.82% | 49 | 20 | RYR2 | p.D2970Y |
| S0000998 | chr2 | 179603942 | C | T | 41.79% | 67 | 28 | TTN | p.G4673E |
| S0000998 | chr2 | 179605753 | C | A | 35.71% | 56 | 20 | TTN | p.Q4069H |
| S0000998 | chr4 | 114158167 | G | C | 33.96% | 53 | 18 | ANK2 | p.A170P |
| S0000998 | chr4 | 190878626 | G | A | 6.30% | 254 | 16 | FRG1 | p.S169N |
| S0000998 | chr8 | 52733231 | G | A | 18.75% | 16 | 3 | PCMTD1 | p.R252* |
| S0000998 | chr8 | 113304801 | T | C | 39.74% | 78 | 31 | CSMD3 | p.N2918S |
| S0000998 | chr12 | 4735742 | C | T | 22.58% | 62 | 14 | AKAP3 | p.V776I |
| S0000998 | chr15 | 33855149 | C | A | 42.86% | 63 | 27 | RYR3 | p.L362M |
| S0000998 | chr16 | 76587243 | C | A | 31.25% | 128 | 40 | CNTNAP4 | p.S1172Y |
| S0000998 | chr2 | 186672199 | GC | G | 21.43% | 28 | 6 | FSIP2 | p.Ala6145fs |
| S0000999 | chr11 | 48387683 | GT | G | 29.09% | 55 | 16 | OR4C5 | p.Thr112fs |
| S0000999 | chr16 | 76573742 | TTA | T | 8.33% | 48 | 4 | CNTNAP4 | p.Ile1120fs |
| S0001000 | chr17 | 7578524 | G | A | 33.16% | 196 | 65 | TP53 | p.Q136* |
| S0001000 | chr11 | 48387683 | GT | G | 26.14% | 88 | 23 | OR4C5 | p.Thr112fs |
| S0001000 | chr11 | 104878040 | GT | G | 9.09% | 44 | 4 | CASP5 | p.Thr81fs |
| S0001000 | chr19 | 1430252 | A | AG | 11.64% | 146 | 17 | DAZAP1 | p.Ala255fs |
| S0001000 | chr20 | 29628225 | AG | A | 5.59% | 286 | 16 | FRG1B | p.Met79fs |
| S0001001 | chr1 | 215813953 | C | T | 8.33% | 36 | 3 | USH2A | p.R4972H |
| S0001001 | chr1 | 216496844 | C | A | 7.81% | 64 | 5 | USH2A | p.A508S |
| S0001001 | chr1 | 237713899 | G | A | 6.00% | 50 | 3 | RYR2 | p.R1039Q |
| S0001001 | chr1 | 237947085 | G | A | 7.32% | 41 | 3 | RYR2 | p.D4031N |
| S0001001 | chr2 | 179587548 | C | T | 10.34% | 29 | 3 | TTN | p.D7360N |
| S0001001 | chr2 | 179611385 | C | T | 8.16% | 49 | 4 | TTN | p.G5248R |
| S0001001 | chr4 | 79432614 | G | A | 13.04% | 23 | 3 | FRAS1 | p.A3323T |
| S0001001 | chr4 | 114276315 | G | A | 5.45% | 55 | 3 | ANK2 | p.D2181N |
| S0001001 | chr6 | 136599903 | C | T | 5.77% | 52 | 3 | BCLAF1 | p.R39H |
| S0001001 | chr7 | 55259515 | T | G | 41.18% | 34 | 14 | EGFR | p.L858R |
| S0001001 | chr7 | 151845367 | G | A | 7.50% | 40 | 3 | KMT2C | p.R4606C |
| S0001001 | chr7 | 151845390 | C | T | 7.14% | 42 | 3 | KMT2C | p.R4598Q |
| S0001001 | chr7 | 151845829 | C | T | 8.57% | 35 | 3 | KMT2C | p.V4452M |
| S0001001 | chr7 | 151853376 | G | A | 7.14% | 42 | 3 | KMT2C | p.P3966L |
| S0001001 | chr7 | 151860427 | C | T | 5.26% | 57 | 3 | KMT2C | p.R3412Q |
| S0001001 | chr7 | 151945256 | G | A | 5.48% | 73 | 4 | KMT2C | p.Q755* |
| S0001001 | chr8 | 12960306 | C | T | 6.98% | 43 | 3 | DLC1 | p.R520Q |
| S0001001 | chr8 | 52733228 | G | A | 9.09% | 33 | 3 | PCMTD1 | p.R253C |
| S0001001 | chr8 | 113266549 | G | A | 5.36% | 56 | 3 | CSMD3 | p.T3348I |
| S0001001 | chr8 | 113563055 | C | T | 6.82% | 44 | 3 | CSMD3 | p.R1470Q |
| S0001001 | chr8 | 113702157 | C | T | 32.00% | 50 | 16 | CSMD3 | p.E699K |
| S0001001 | chr8 | 113988234 | C | T | 5.77% | 52 | 3 | CSMD3 | p.E392K |
| S0001001 | chr8 | 134042152 | G | A | 11.43% | 35 | 4 | TG | p.G2375R |
| S0001001 | chr12 | 4736759 | C | T | 5.66% | 53 | 3 | AKAP3 | p.E437K |
| S0001001 | chr17 | 7577548 | C | T | 13.24% | 68 | 9 | TP53 | p.G245S |
| S0001001 | chr19 | 33490585 | G | A | 17.50% | 40 | 7 | RHPN2 | p.Q378* |
| S0001001 | chr20 | 29625934 | C | T | 10.00% | 50 | 5 | FRG1B | p.H60Y |
| S0001001 | chr20 | 29628263 | A | G | 5.85% | 171 | 10 | FRG1B | p.I89V |
| S0001001 | chr1 | 145014186 | GA | G | 22.78% | 79 | 18 | PDE4DIP | p.Ser119fs |
| S0001001 | chr17 | 7565292 | T | TG | 9.38% | 32 | 3 | TP53 | p.Ser275fs |
| S0001001 | chr20 | 29625924 | A | ACTT | 9.43% | 106 | 10 | FRG1B | p.Leu57dup |
| S0001029 | chr1 | 237656366 | G | A | 31.03% | 58 | 18 | RYR2 | p.R645H |
| S0001029 | chr8 | 52733079 | A | C | 8.57% | 35 | 3 | PCMTD1 | p.S302R |
| S0001029 | chr4 | 190882977 | AAAG | A | 13.04% | 23 | 3 | FRG1 | p.Lys212del |
| S0001031 | chr1 | 237538068 | G | T | 6.90% | 87 | 6 | RYR2 | p.D144Y |
| S0001031 | chr2 | 141215070 | G | A | 6.82% | 44 | 3 | LRP1B | p.T3259I |
| S0001031 | chr2 | 179434243 | G | T | 6.06% | 66 | 4 | TTN | p.T25539K |
| S0001031 | chr2 | 179441391 | C | A | 6.32% | 95 | 6 | TTN | p.V23194F |
| S0001031 | chr7 | 151945334 | T | C | 8.11% | 74 | 6 | KMT2C | p.N729D |
| S0001031 | chr7 | 151962289 | C | T | 5.30% | 151 | 8 | KMT2C | p.E340K |
| S0001031 | chr8 | 13356639 | A | T | 6.41% | 78 | 5 | DLC1 | p.D314E |
| S0001031 | chr4 | 190876218 | A | AAT | 7.32% | 82 | 6 | FRG1 | p.Leu117fs |
| S0001031 | chr12 | 4737628 | ATCT | A | 6.06% | 66 | 4 | AKAP3 | p.Lys146del |
| S0001033 | chr4 | 190878577 | A | G | 5.41% | 185 | 10 | FRG1 | p.N153D |
| S0001033 | chr19 | 33490500 | C | T | 6.78% | 59 | 4 | RHPN2 | p.R406Q |
| S0001033 | chr11 | 48387683 | GT | G | 24.42% | 86 | 21 | OR4C5 | p.Thr112fs |
| S0001033 | chr19 | 1430252 | A | AGG | 11.11% | 36 | 4 | DAZAP1 | p.Ala255fs |
| S0001033 | chr20 | 29632611 | AAAG | A | 10.45% | 134 | 14 | FRG1B | p.Lys144del |
| S0001035 | chr2 | 179584152 | G | A | 30.00% | 20 | 6 | TTN | p.R7989C |
| S0001035 | chr2 | 179588751 | G | A | 6.52% | 46 | 3 | TTN | p.H7079Y |
| S0001035 | chr3 | 195515594 | C | G | 13.04% | 46 | 6 | MUC4 | p.E953Q |
| S0001035 | chr3 | 195515617 | G | C | 10.87% | 46 | 5 | MUC4 | p.T945S |
| S0001035 | chr3 | 195517258 | G | A | 17.78% | 45 | 8 | MUC4 | p.T398I |
| S0001035 | chr3 | 195517321 | G | A | 22.00% | 50 | 11 | MUC4 | p.T377I |
| S0001035 | chr3 | 195517553 | A | C | 20.59% | 68 | 14 | MUC4 | p.F300V |
| S0001035 | chr4 | 114277429 | C | T | 7.32% | 41 | 3 | ANK2 | p.P2552L |
| S0001035 | chr7 | 151860454 | C | T | 5.56% | 54 | 3 | KMT2C | p.R3403H |
| S0001035 | chr8 | 13356802 | G | A | 18.92% | 37 | 7 | DLC1 | p.T260I |
| S0001035 | chr8 | 13356819 | T | G | 16.22% | 37 | 6 | DLC1 | p.Q254H |
| S0001035 | chr8 | 133984058 | C | T | 9.68% | 62 | 6 | TG | p.R1999W |
| S0001035 | chr2 | 179480406 | G | GA | 5.88% | 51 | 3 | TTN | p.Ala16141fs |
| S0001035 | chr2 | 179480412 | TC | T | 5.77% | 52 | 3 | TTN | p.Glu16139fs |
| S0001035 | chr4 | 88035654 | A | AAC | 15.00% | 20 | 3 | AFF1 | p.Ser557fs |
| S0001035 | chr8 | 13356789 | GT | G | 6.82% | 44 | 3 | DLC1 | p.Asn264fs |
| S0001035 | chr11 | 48387284 | G | GAGTCTTTAGTA | 10.71% | 28 | 3 | OR4C5 | p.Ser245fs |
| S0001035 | chr15 | 23686067 | TCTC | T | 7.46% | 67 | 5 | GOLGA6L2 | p.Glu518del |
| S0001035 | chr20 | 29625925 | C | CTTG | 10.00% | 100 | 10 | FRG1B | p.Val58dup |
| S0001037 | chr7 | 151945334 | T | C | 5.49% | 91 | 5 | KMT2C | p.N729D |
| S0001037 | chr17 | 7578190 | T | C | 36.69% | 139 | 51 | TP53 | p.Y220C |
| S0001037 | chr7 | 55242464 | AGGAATTAAGAGAAGC | A | 11.90% | 84 | 10 | EGFR | p.Glu746_Ala750del |
| S0001037 | chr11 | 48387683 | GT | G | 23.68% | 38 | 9 | OR4C5 | p.Thr112fs |
| S0001038 | chr1 | 237756920 | G | A | 5.08% | 59 | 3 | RYR2 | p.G1472R |
| S0001038 | chr2 | 179514029 | C | T | 5.36% | 56 | 3 | TTN | p.V13335I |
| S0001038 | chr2 | 179547565 | G | A | 9.38% | 32 | 3 | TTN | p.R10985W |
| S0001038 | chr2 | 179638835 | G | A | 5.56% | 72 | 4 | TTN | p.R2354C |
| S0001038 | chr2 | 186603394 | G | A | 6.00% | 50 | 3 | FSIP2 | p.G14R |
| S0001038 | chr4 | 190884267 | G | A | 7.14% | 42 | 3 | FRG1 | p.D254N |
| S0001038 | chr8 | 113267591 | G | A | 6.98% | 43 | 3 | CSMD3 | p.Q3310* |
| S0001038 | chr8 | 113599308 | C | G | 6.67% | 45 | 3 | CSMD3 | p.G1291A |
| S0001038 | chr8 | 133978868 | C | A | 10.91% | 55 | 6 | TG | p.S1871* |
| S0001038 | chr12 | 4736113 | C | T | 6.38% | 47 | 3 | AKAP3 | p.G652E |
| S0001038 | chr12 | 57111404 | A | G | 6.67% | 45 | 3 | NACA | p.S1304P |
| S0001038 | chr12 | 57111493 | A | G | 7.89% | 38 | 3 | NACA | p.L1274P |
| S0001038 | chr14 | 32615491 | C | T | 7.50% | 40 | 3 | ARHGAP5 | p.T124I |
| S0001038 | chr7 | 55242464 | AGGAATTAAGAGAAGC | A | 10.29% | 68 | 7 | EGFR | p.Glu746_Ala750del |
| S0001038 | chr12 | 49434939 | GC | G | 6.98% | 43 | 3 | KMT2D | p.Ala2205fs |
| S0001039 | chr8 | 101719201 | A | G | 6.25% | 48 | 3 | PABPC1 | p.I454T |
| S0001039 | chr4 | 190878551 | AG | A | 8.06% | 62 | 5 | FRG1 | p.Met147fs |
| S0001039 | chr11 | 48387284 | G | GAGTCTTTAGTA | 12.90% | 31 | 4 | OR4C5 | p.Ser245fs |
| S0001039 | chr20 | 29625899 | A | AAT | 5.36% | 56 | 3 | FRG1B | p.Leu50fs |
| S0001039 | chr20 | 29625924 | A | ACTT | 6.38% | 47 | 3 | FRG1B | p.Leu57dup |
| S0001039 | chr20 | 29628229 | G | GA | 7.89% | 114 | 9 | FRG1B | p.Met79fs |
| S0001040 | chr4 | 114280179 | G | T | 8.33% | 36 | 3 | ANK2 | p.D3469Y |
| S0001040 | chr8 | 101719201 | A | G | 11.29% | 62 | 7 | PABPC1 | p.I454T |
| S0001040 | chr19 | 33490500 | C | T | 13.33% | 60 | 8 | RHPN2 | p.R406Q |
| S0001040 | chr2 | 186678399 | AT | A | 5.66% | 53 | 3 | FSIP2 | p.Leu6742fs |
| S0001040 | chr7 | 55242468 | ATTAAGAGAAGCAACATCT | A | 18.60% | 43 | 8 | EGFR | p.Leu747_Ser752del |
| S0001040 | chr11 | 48387285 | A | AGTCTTTAGTAG | 6.90% | 58 | 4 | OR4C5 | p.Ser245fs |
| S0001040 | chr19 | 1430252 | A | AG | 13.64% | 22 | 3 | DAZAP1 | p.Ala255fs |
| S0001043 | chr1 | 144854581 | T | C | 12.50% | 32 | 4 | PDE4DIP | p.T2433A |
| S0001043 | chr1 | 237791224 | T | C | 8.33% | 36 | 3 | RYR2 | p.I2093T |
| S0001043 | chr2 | 179476821 | C | T | 14.29% | 21 | 3 | TTN | p.E16773K |
| S0001043 | chr2 | 186659391 | G | A | 8.33% | 36 | 3 | FSIP2 | p.E2599K |
| S0001043 | chr4 | 114179246 | G | A | 7.69% | 39 | 3 | ANK2 | p.R410H |
| S0001043 | chr4 | 114280150 | C | A | 10.34% | 29 | 3 | ANK2 | p.P3459H |
| S0001043 | chr8 | 12957475 | C | T | 8.93% | 56 | 5 | DLC1 | p.V791M |
| S0001043 | chr8 | 77764207 | G | A | 8.33% | 72 | 6 | ZFHX4 | p.A1684T |
| S0001043 | chr8 | 113841991 | C | T | 7.14% | 42 | 3 | CSMD3 | p.D595N |
| S0001043 | chr12 | 49431832 | C | T | 7.69% | 52 | 4 | KMT2D | p.A3103T |
| S0001043 | chr19 | 21476519 | T | C | 8.20% | 61 | 5 | ZNF708 | p.K417E |
| S0001043 | chr19 | 39051877 | G | A | 7.69% | 52 | 4 | RYR1 | p.R4136H |
| S0001043 | chr2 | 179544685 | CTCT | C | 5.88% | 51 | 3 | TTN | p.Glu11172del |
| S0001044 | chr2 | 179440330 | A | G | 6.56% | 61 | 4 | TTN | p.M23510T |
| S0001044 | chr8 | 113988228 | G | C | 7.35% | 68 | 5 | CSMD3 | p.Q394E |
| S0001044 | chr8 | 134030152 | T | C | 5.34% | 206 | 11 | TG | p.V2231A |
| S0001044 | chr17 | 7577539 | G | A | 20.00% | 85 | 17 | TP53 | p.R248W |
| S0001044 | chr7 | 55242465 | GGAATTAAGA | G | 20.29% | 69 | 14 | EGFR | p.Leu747_Glu749del |
| S0001044 | chr17 | 7565292 | T | TG | 13.64% | 22 | 3 | TP53 | p.Ser275fs |
| S0001045 | chr1 | 145014096 | G | A | 11.76% | 34 | 4 | PDE4DIP | p.A149V |
| S0001045 | chr4 | 79442734 | G | A | 11.11% | 27 | 3 | FRAS1 | p.R3533Q |
| S0001045 | chr4 | 190876218 | A | AAT | 5.45% | 55 | 3 | FRG1 | p.Leu117fs |
| S0001045 | chr19 | 1430252 | A | AG | 13.22% | 121 | 16 | DAZAP1 | p.Ala255fs |

Supplementary Table 2. Genetic variants detected in elderly lung adenocarcinoma patients.

Patient ID (patient_ID), chromosome number (chr), position number (position), reference allele (ref_allele), altered allele (alt_allele), the percentage of variant (fre), total sequencing depth in the corresponding genetic position (dp), detected sequencing depth of the variant allele (alt_dp), the gene where the variant locate (gene) and the corresponding amino acid alteration (mutation_p) are provided.

| **patient_ID** | **chr** | **position** | **ref_allele** | **alt_allele** | **fre** | **dp** | **alt_dp** | **gene** | **mutation_p** |
| --- | --- | --- | --- | --- | --- | --- | --- | --- | --- |
| S0000121 | chr1 | 75065486 | G | T | 10.42% | 96 | 10 | C1orf173 | p.P540H |
| S0000121 | chr1 | 216496838 | C | A | 29.55% | 88 | 26 | USH2A | p.D510Y |
| S0000121 | chr1 | 237433807 | T | A | 15.38% | 78 | 12 | RYR2 | p.W18R |
| S0000121 | chr1 | 237944913 | G | A | 38.46% | 26 | 10 | RYR2 | p.D3983N |
| S0000121 | chr1 | 237947275 | A | G | 15.32% | 111 | 17 | RYR2 | p.H4094R |
| S0000121 | chr2 | 141474358 | A | G | 7.77% | 103 | 8 | LRP1B | p.M1929T |
| S0000121 | chr2 | 141665476 | G | T | 7.14% | 84 | 6 | LRP1B | p.P1164T |
| S0000121 | chr2 | 141680567 | C | G | 17.95% | 78 | 14 | LRP1B | p.D1096H |
| S0000121 | chr2 | 179416636 | G | T | 19.05% | 84 | 16 | TTN | p.P30331T |
| S0000121 | chr2 | 179468967 | G | C | 9.38% | 64 | 6 | TTN | p.I18149M |
| S0000121 | chr2 | 179475981 | G | T | 16.13% | 62 | 10 | TTN | p.L16959M |
| S0000121 | chr2 | 179575503 | C | A | 12.20% | 41 | 5 | TTN | p.G9441* |
| S0000121 | chr2 | 179593431 | C | A | 15.70% | 121 | 19 | TTN | p.V6408L |
| S0000121 | chr2 | 186655247 | C | A | 26.58% | 79 | 21 | FSIP2 | p.H1217Q |
| S0000121 | chr2 | 186658662 | G | A | 10.26% | 39 | 4 | FSIP2 | p.E2356K |
| S0000121 | chr3 | 195516534 | G | T | 13.85% | 65 | 9 | MUC4 | p.H639Q |
| S0000121 | chr3 | 195517277 | T | A | 8.51% | 47 | 4 | MUC4 | p.K392* |
| S0000121 | chr6 | 136594292 | T | C | 10.75% | 93 | 10 | BCLAF1 | p.N629S |
| S0000121 | chr7 | 151873605 | C | A | 7.78% | 90 | 7 | KMT2C | p.R2978M |
| S0000121 | chr8 | 52733231 | G | A | 13.04% | 23 | 3 | PCMTD1 | p.R252* |
| S0000121 | chr8 | 77620043 | C | A | 24.47% | 94 | 23 | ZFHX4 | p.N951K |
| S0000121 | chr8 | 77764334 | C | T | 9.28% | 97 | 9 | ZFHX4 | p.P1726L |
| S0000121 | chr8 | 113599373 | A | C | 23.40% | 47 | 11 | CSMD3 | p.Y1269* |
| S0000121 | chr8 | 134125675 | G | T | 11.36% | 88 | 10 | TG | p.E2528* |
| S0000121 | chr12 | 49432079 | C | A | 26.44% | 87 | 23 | KMT2D | p.K3020N |
| S0000121 | chr14 | 32561340 | G | A | 7.50% | 40 | 3 | ARHGAP5 | p.E489K |
| S0000121 | chr15 | 33765632 | G | A | 11.11% | 81 | 9 | RYR3 | p.V22I |
| S0000121 | chr15 | 34118468 | G | C | 16.18% | 68 | 11 | RYR3 | p.R3721P |
| S0000121 | chr19 | 21607526 | A | G | 7.14% | 42 | 3 | ZNF493 | p.K689E |
| S0000121 | chr19 | 39001324 | T | C | 20.22% | 89 | 18 | RYR1 | p.Y3009H |
| S0000121 | chr19 | 39009925 | C | T | 8.42% | 95 | 8 | RYR1 | p.R3364W |
| S0000121 | chr1 | 152129065 | GTGGTGGGAATCTCTGTCTTGTTTCTCAGACTGACCA | G | 26.60% | 188 | 50 | RPTN | p.Gly159_His170del |
| S0000121 | chr8 | 101721932 | CT | C | 14.47% | 76 | 11 | PABPC1 | p.Phe335fs |
| S0000121 | chr11 | 104878040 | GT | G | 6.52% | 46 | 3 | CASP5 | p.Thr81fs |
| S0000121 | chr17 | 7577053 | AG | A | 31.82% | 66 | 21 | TP53 | p.Pro295fs |
| S0000121 | chr19 | 21492093 | CT | C | 16.04% | 106 | 17 | ZNF708 | p.Glu61fs |
| S0000237 | chr4 | 114120261 | C | A | 5.98% | 117 | 7 | ANK2 | p.S127Y |
| S0000237 | chr4 | 114279456 | G | A | 11.43% | 140 | 16 | ANK2 | p.V3228M |
| S0000237 | chr8 | 52733231 | G | A | 9.09% | 44 | 4 | PCMTD1 | p.R252* |
| S0000237 | chr19 | 21607526 | A | G | 5.32% | 94 | 5 | ZNF493 | p.K689E |
| S0000237 | chr4 | 88035720 | C | CG | 5.08% | 59 | 3 | AFF1 | p.Ala580fs |
| S0000272 | chr6 | 136597006 | G | C | 6.90% | 87 | 6 | BCLAF1 | p.P553A |
| S0000272 | chr8 | 133899740 | C | A | 7.89% | 38 | 3 | TG | p.A708D |
| S0000272 | chr11 | 4976768 | C | T | 6.52% | 46 | 3 | OR51A2 | p.G59E |
| S0000272 | chr1 | 152129065 | GTGGTGGGAATCTCTGTCTTGTTTCTCAGACTGACCA | G | 22.17% | 230 | 51 | RPTN | p.Gly159_His170del |
| S0000272 | chr4 | 88035714 | G | GC | 5.17% | 58 | 3 | AFF1 | p.Arg579fs |
| S0000277 | chr7 | 55259515 | T | G | 16.85% | 89 | 15 | EGFR | p.L858R |
| S0000277 | chr4 | 88035714 | G | GC | 5.77% | 52 | 3 | AFF1 | p.Arg579fs |
| S0000283 | chr14 | 32561340 | G | A | 12.82% | 78 | 10 | ARHGAP5 | p.E489K |
| S0000283 | chr15 | 33831614 | G | A | 41.67% | 36 | 15 | RYR3 | p.R166Q |
| S0000283 | chr7 | 55242466 | GAATTAAGAGAAGCAACAT | G | 25.60% | 125 | 32 | EGFR | p.Glu746_Ser752delinsA |
| S0000283 | chr15 | 23686045 | A | ATCT | 12.00% | 50 | 6 | GOLGA6L2 | p.Glu525_Met526insK |
| S0000283 | chr17 | 7577571 | ATGTAGT | A | 50.00% | 46 | 23 | TP53 | p.Asn235_Tyr236del |
| S0000284 | chr1 | 216144041 | C | A | 13.89% | 72 | 10 | USH2A | p.G2295* |
| S0000284 | chr4 | 79238606 | G | A | 8.96% | 67 | 6 | FRAS1 | p.G635D |
| S0000284 | chr8 | 12946158 | A | G | 12.28% | 57 | 7 | DLC1 | p.V1377A |
| S0000284 | chr8 | 52733242 | C | T | 6.52% | 46 | 3 | PCMTD1 | p.R248H |
| S0000284 | chr8 | 77763893 | C | A | 10.56% | 161 | 17 | ZFHX4 | p.P1579H |
| S0000284 | chr11 | 4976768 | C | T | 5.19% | 77 | 4 | OR51A2 | p.G59E |
| S0000284 | chr19 | 33490500 | C | T | 16.00% | 25 | 4 | RHPN2 | p.R406Q |
| S0000284 | chr1 | 152129065 | GTGGTGGGAATCTCTGTCTTGTTTCTCAGACTGACCA | G | 35.20% | 179 | 63 | RPTN | p.Gly159_His170del |
| S0000284 | chr8 | 113668486 | GC | G | 11.11% | 162 | 18 | CSMD3 | p.Gly967fs |
| S0000285 | chr6 | 136594292 | T | C | 6.03% | 116 | 7 | BCLAF1 | p.N629S |
| S0000285 | chr19 | 21607526 | A | G | 5.09% | 275 | 14 | ZNF493 | p.K689E |
| S0000285 | chr7 | 55242464 | AGGAATTAAGAGAAGC | A | 10.00% | 110 | 11 | EGFR | p.Glu746_Ala750del |
| S0000285 | chr15 | 23686010 | T | TCTC | 16.67% | 72 | 12 | GOLGA6L2 | p.Glu537dup |
| S0000291 | chr1 | 75038720 | C | A | 14.48% | 145 | 21 | C1orf173 | p.A892S |
| S0000291 | chr1 | 237817641 | G | A | 21.25% | 80 | 17 | RYR2 | p.G2629D |
| S0000291 | chr2 | 141202159 | C | T | 12.37% | 97 | 12 | LRP1B | p.G3383R |
| S0000291 | chr2 | 179414070 | C | G | 11.11% | 117 | 13 | TTN | p.K30761N |
| S0000291 | chr2 | 179443931 | A | T | 27.27% | 77 | 21 | TTN | p.I22609K |
| S0000291 | chr7 | 104753671 | G | T | 29.93% | 147 | 44 | KMT2E | p.G1823V |
| S0000291 | chr8 | 113504808 | C | A | 34.04% | 94 | 32 | CSMD3 | p.G1730C |
| S0000291 | chr12 | 57112976 | T | C | 23.53% | 68 | 16 | NACA | p.T780A |
| S0000291 | chr15 | 23686515 | C | A | 17.07% | 41 | 7 | GOLGA6L2 | p.E369D |
| S0000291 | chr20 | 29630703 | C | T | 9.40% | 149 | 14 | FRG1B | p.S74F |
| S0000291 | chr17 | 7576880 | TG | T | 51.75% | 114 | 59 | TP53 | p.Pro322fs |
| S0000298 | chr1 | 144930880 | C | A | 9.09% | 143 | 13 | PDE4DIP | p.D277Y |
| S0000298 | chr6 | 136594292 | T | C | 9.72% | 72 | 7 | BCLAF1 | p.N629S |
| S0000298 | chr7 | 55259515 | T | G | 16.67% | 120 | 20 | EGFR | p.L858R |
| S0000298 | chr8 | 52733242 | C | T | 8.82% | 34 | 3 | PCMTD1 | p.R248H |
| S0000298 | chr19 | 1430252 | A | AG | 13.64% | 44 | 6 | DAZAP1 | p.Ala255fs |
| S0000299 | chr1 | 237550611 | G | A | 9.17% | 109 | 10 | RYR2 | p.V201M |
| S0000299 | chr7 | 55259515 | T | G | 21.21% | 99 | 21 | EGFR | p.L858R |
| S0000299 | chr8 | 52733228 | G | A | 12.50% | 32 | 4 | PCMTD1 | p.R253C |
| S0000299 | chr8 | 52733231 | G | A | 10.34% | 29 | 3 | PCMTD1 | p.R252* |
| S0000299 | chr11 | 4976768 | C | T | 6.45% | 62 | 4 | OR51A2 | p.G59E |
| S0000299 | chr14 | 32561340 | G | A | 8.82% | 34 | 3 | ARHGAP5 | p.E489K |
| S0000299 | chr11 | 104878040 | GT | G | 8.62% | 58 | 5 | CASP5 | p.Thr81fs |
| S0000300 | chr2 | 179613382 | T | C | 16.13% | 93 | 15 | TTN | p.Q4582R |
| S0000300 | chr7 | 55259515 | T | G | 19.00% | 100 | 19 | EGFR | p.L858R |
| S0000300 | chr8 | 77764245 | G | T | 14.39% | 139 | 20 | ZFHX4 | p.M1696I |
| S0000300 | chr17 | 7577548 | C | A | 30.16% | 63 | 19 | TP53 | p.G245C |
| S0000300 | chr15 | 23686067 | TCTC | T | 10.34% | 58 | 6 | GOLGA6L2 | p.Glu518del |
| S0000300 | chr19 | 1430252 | A | AG | 18.18% | 44 | 8 | DAZAP1 | p.Ala255fs |
| S0000303 | chr1 | 152129087 | T | C | 30.88% | 285 | 88 | RPTN | p.K163R |
| S0000303 | chr1 | 152129094 | A | G | 31.51% | 292 | 92 | RPTN | p.S161P |
| S0000303 | chr1 | 152129115 | T | C | 14.05% | 299 | 42 | RPTN | p.R154G |
| S0000303 | chr14 | 32561340 | G | A | 12.50% | 48 | 6 | ARHGAP5 | p.E489K |
| S0000303 | chr4 | 88035714 | G | GC | 10.64% | 47 | 5 | AFF1 | p.Arg579fs |
| S0000303 | chr7 | 55242464 | AGGAATTAAGAGAAGC | A | 48.48% | 198 | 96 | EGFR | p.Glu746_Ala750del |
| S0000303 | chr15 | 23686067 | TCTC | T | 5.13% | 78 | 4 | GOLGA6L2 | p.Glu518del |
| S0000303 | chr17 | 7578463 | CGGGTG | C | 39.53% | 86 | 34 | TP53 | p.Thr155fs |
| S0000307 | chr2 | 140995765 | C | T | 17.39% | 69 | 12 | LRP1B | p.D4506N |
| S0000307 | chr8 | 113418918 | G | A | 29.76% | 84 | 25 | CSMD3 | p.P1882S |
| S0000307 | chr12 | 57112033 | G | A | 6.90% | 58 | 4 | NACA | p.P1094L |
| S0000307 | chr17 | 7578479 | G | A | 38.24% | 68 | 26 | TP53 | p.P151S |
| S0000307 | chr19 | 33490500 | C | T | 6.35% | 126 | 8 | RHPN2 | p.R406Q |
| S0000307 | chr4 | 88035714 | G | GC | 7.69% | 39 | 3 | AFF1 | p.Arg579fs |
| S0000320 | chr1 | 237947200 | C | T | 9.84% | 122 | 12 | RYR2 | p.T4069M |
| S0000320 | chr7 | 55241677 | G | A | 35.96% | 89 | 32 | EGFR | p.E709K |
| S0000320 | chr7 | 55241707 | G | A | 37.18% | 78 | 29 | EGFR | p.G719S |
| S0000320 | chr8 | 52733079 | A | C | 9.30% | 43 | 4 | PCMTD1 | p.S302R |
| S0000320 | chr8 | 52733242 | C | T | 11.11% | 36 | 4 | PCMTD1 | p.R248H |
| S0000320 | chr17 | 7576897 | G | A | 15.38% | 78 | 12 | TP53 | p.Q317* |
| S0000320 | chr11 | 104878040 | G | GT | 6.38% | 47 | 3 | CASP5 | p.Thr81fs |
| S0000321 | chr2 | 179480409 | G | A | 23.40% | 47 | 11 | TTN | p.P16140L |
| S0000321 | chr4 | 190878577 | A | G | 6.94% | 216 | 15 | FRG1 | p.N153D |
| S0000321 | chr6 | 136594292 | T | C | 8.11% | 37 | 3 | BCLAF1 | p.N629S |
| S0000321 | chr7 | 55259515 | T | G | 29.87% | 77 | 23 | EGFR | p.L858R |
| S0000321 | chr8 | 52733079 | A | C | 13.16% | 38 | 5 | PCMTD1 | p.S302R |
| S0000321 | chr8 | 101719201 | A | G | 6.10% | 82 | 5 | PABPC1 | p.I454T |
| S0000321 | chr8 | 101721932 | CT | C | 29.63% | 54 | 16 | PABPC1 | p.Phe335fs |
| S0000321 | chr15 | 23686088 | TCTC | T | 21.95% | 82 | 18 | GOLGA6L2 | p.Glu511del |
| S0000323 | chr2 | 141747206 | C | A | 9.09% | 77 | 7 | LRP1B | p.D889Y |
| S0000323 | chr19 | 33490500 | C | T | 11.40% | 114 | 13 | RHPN2 | p.R406Q |
| S0000323 | chr1 | 152129065 | GTGGTGGGAATCTCTGTCTTGTTTCTCAGACTGACCA | G | 28.95% | 190 | 55 | RPTN | p.Gly159_His170del |
| S0000323 | chr4 | 190878551 | AG | A | 11.59% | 69 | 8 | FRG1 | p.Met147fs |
| S0000323 | chr4 | 190878555 | G | GA | 5.71% | 70 | 4 | FRG1 | p.Met147fs |
| S0000323 | chr7 | 55242464 | AGGAATTAAGAGAAGC | A | 42.31% | 78 | 33 | EGFR | p.Glu746_Ala750del |
| S0000323 | chr20 | 29625924 | A | ACTT | 10.42% | 96 | 10 | FRG1B | p.Leu57dup |
| S0000346 | chr7 | 55259515 | T | G | 12.05% | 83 | 10 | EGFR | p.L858R |
| S0000346 | chr12 | 57112273 | A | G | 5.08% | 59 | 3 | NACA | p.V1014A |
| S0000346 | chr17 | 7577534 | C | A | 20.34% | 59 | 12 | TP53 | p.R249S |
| S0000346 | chr19 | 33490500 | C | T | 8.26% | 109 | 9 | RHPN2 | p.R406Q |
| S0000347 | chr7 | 55259515 | T | G | 34.07% | 91 | 31 | EGFR | p.L858R |
| S0000347 | chr11 | 104879686 | CT | C | 6.38% | 94 | 6 | CASP5 | p.Arg23fs |
| S0000424 | chr1 | 75097578 | A | G | 6.25% | 64 | 4 | C1orf173 | p.I213T |
| S0000424 | chr7 | 55259515 | T | G | 20.25% | 79 | 16 | EGFR | p.L858R |
| S0000424 | chr11 | 4976768 | C | T | 6.78% | 59 | 4 | OR51A2 | p.G59E |
| S0000424 | chr19 | 21607526 | A | G | 6.06% | 99 | 6 | ZNF493 | p.K689E |
| S0000440 | chr1 | 216052344 | C | A | 21.54% | 130 | 28 | USH2A | p.A2774S |
| S0000440 | chr1 | 237881816 | G | A | 22.06% | 68 | 15 | RYR2 | p.G3515S |
| S0000440 | chr2 | 141458107 | C | A | 16.28% | 86 | 14 | LRP1B | p.A2171S |
| S0000440 | chr2 | 179632752 | C | A | 11.93% | 109 | 13 | TTN | p.Q3098H |
| S0000440 | chr2 | 186604154 | C | A | 7.89% | 76 | 6 | FSIP2 | p.P150T |
| S0000440 | chr6 | 136590698 | C | T | 9.62% | 52 | 5 | BCLAF1 | p.R699H |
| S0000440 | chr8 | 77616611 | G | A | 9.22% | 141 | 13 | ZFHX4 | p.M96I |
| S0000440 | chr14 | 32561340 | G | A | 13.16% | 38 | 5 | ARHGAP5 | p.E489K |
| S0000440 | chr15 | 33954884 | T | C | 8.33% | 156 | 13 | RYR3 | p.V1718A |
| S0000440 | chr15 | 34135738 | T | C | 12.68% | 71 | 9 | RYR3 | p.V4420A |
| S0000440 | chr17 | 7578457 | C | A | 20.00% | 90 | 18 | TP53 | p.R158L |
| S0000440 | chr19 | 38993295 | G | A | 8.48% | 165 | 14 | RYR1 | p.R2588H |
| S0000440 | chr2 | 179600746 | AC | A | 8.57% | 70 | 6 | TTN | p.Gly4809fs |
| S0000440 | chr4 | 190876218 | A | AAT | 5.43% | 129 | 7 | FRG1 | p.Leu117fs |
| S0000440 | chr11 | 48387683 | GT | G | 35.11% | 94 | 33 | OR4C5 | p.Thr112fs |
| S0000441 | chr7 | 104753551 | G | A | 15.65% | 147 | 23 | KMT2E | p.G1783E |
| S0000473 | chr1 | 75037744 | G | A | 28.57% | 49 | 14 | C1orf173 | p.A1217V |
| S0000473 | chr1 | 75037745 | C | A | 26.53% | 49 | 13 | C1orf173 | p.A1217S |
| S0000473 | chr1 | 215901582 | C | G | 7.55% | 53 | 4 | USH2A | p.E3952D |
| S0000473 | chr1 | 237948149 | C | G | 15.91% | 44 | 7 | RYR2 | p.I4385M |
| S0000473 | chr2 | 141458182 | C | T | 20.59% | 34 | 7 | LRP1B | p.V2146I |
| S0000473 | chr7 | 104730560 | G | A | 10.81% | 37 | 4 | KMT2E | p.R488K |
| S0000473 | chr7 | 151878988 | C | A | 7.50% | 80 | 6 | KMT2C | p.R1986L |
| S0000473 | chr8 | 113299388 | C | A | 36.07% | 61 | 22 | CSMD3 | p.R3079L |
| S0000473 | chr12 | 4736152 | C | A | 30.56% | 36 | 11 | AKAP3 | p.R639M |
| S0000473 | chr12 | 57113833 | G | A | 6.78% | 59 | 4 | NACA | p.S494F |
| S0000476 | chr8 | 52733228 | G | A | 9.09% | 33 | 3 | PCMTD1 | p.R253C |
| S0000476 | chr11 | 104878040 | GT | G | 6.25% | 48 | 3 | CASP5 | p.Thr81fs |
| S0000476 | chr15 | 23686067 | TCTC | T | 13.33% | 45 | 6 | GOLGA6L2 | p.Glu518del |
| S0000476 | chr15 | 23686101 | C | CTGCATCTTCTCCTGCTCT | 15.09% | 53 | 8 | GOLGA6L2 | p.Gln507_Glu508insEQEKMQ |
| S0000476 | chr19 | 1430252 | A | AGGC | 11.36% | 44 | 5 | DAZAP1 | p.Gly254dup |
| S0000476 | chr20 | 29625899 | A | AAT | 8.33% | 168 | 14 | FRG1B | p.Leu50fs |
| S0000606 | chr1 | 215848816 | T | A | 8.77% | 57 | 5 | USH2A | p.Q4146L |
| S0000606 | chr2 | 179428532 | G | A | 6.98% | 43 | 3 | TTN | p.R27443C |
| S0000606 | chr6 | 136594292 | T | C | 7.69% | 52 | 4 | BCLAF1 | p.N629S |
| S0000606 | chr7 | 55259515 | T | G | 20.90% | 67 | 14 | EGFR | p.L858R |
| S0000606 | chr17 | 7578403 | C | T | 13.04% | 46 | 6 | TP53 | p.C176Y |
| S0000606 | chr16 | 76572197 | CTTT | C | 6.52% | 46 | 3 | CNTNAP4 | p.Phe1064del |
| S0000606 | chr19 | 1430252 | A | AG | 6.98% | 43 | 3 | DAZAP1 | p.Ala255fs |
